# The BAR Domain Superfamily Proteins from Subcellular Structures to Human Diseases

**DOI:** 10.3390/membranes2010091

**Published:** 2012-02-27

**Authors:** Fatemeh Safari, Shiro Suetsugu

**Affiliations:** Laboratory of Membrane and Cytoskeleton Dynamics, Institute of Molecular and Cellular Biosciences, The University of Tokyo, 1-1-1 Yayoi, Bunkyo-ku, Tokyo 113-0032, Japan

**Keywords:** BAR domain, EFC domain, F-BAR domain, I-BAR domain, N-WASP, WAVE, dynamin, membrane curvature, plasma membrane, endocytosis, endosome, autophagosome, podosome

## Abstract

Eukaryotic cells have complicated membrane systems. The outermost plasma membrane contains various substructures, such as invaginations and protrusions, which are involved in endocytosis and cell migration. Moreover, the intracellular membrane compartments, such as autophagosomes and endosomes, are essential for cellular viability. The Bin-Amphiphysin-Rvs167 (BAR) domain superfamily proteins are important players in membrane remodeling through their structurally determined membrane binding surfaces. A variety of BAR domain superfamily proteins exist, and each family member appears to be involved in the formation of certain subcellular structures or intracellular membrane compartments. Most of the BAR domain superfamily proteins contain SH3 domains, which bind to the membrane scission molecule, dynamin, as well as the actin regulatory WASP/WAVE proteins and several signal transduction molecules, providing possible links between the membrane and the cytoskeleton or other machineries. In this review, we summarize the current information about each BAR superfamily protein with an SH3 domain(s). The involvement of BAR domain superfamily proteins in various diseases is also discussed.

## 1. Introduction

**Figure 1:**
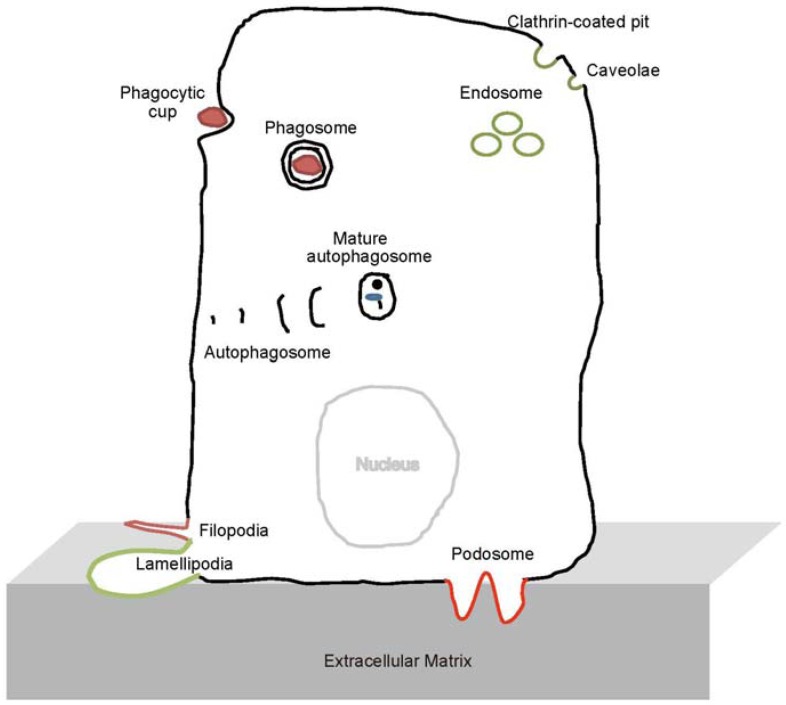
Subcellular structures on which Bin-Amphiphysin-Rvs167 (BAR) domain proteins act, as discussed in this review.

The relationship between cellular morphology and diseases, such as cancer, has been unclear. However, transformed cancer cells are often first recognized by changes in their morphology. The shapes of transformed cancer cells are apparently different from those of the normal parental cells, as observed microscopically. The shape of the eukaryotic plasma membrane changes during various processes, such as cell division, cell movement, and differentiation. Eukaryotic cells also contain various intracellular vesicles that synthesize, traffic, and degrade materials, such as proteins, during membrane remodeling, including the fission and fusion of vesicles (Figure 1). Dynamic remodeling of the membrane is achieved by the interplay between proteins and lipids. Among these proteins, accumulating evidence indicates that the Bin-Amphiphysin-Rvs167 (BAR) domain superfamily proteins (referred to hereafter as BAR proteins) play key roles. 

**Figure 2:**
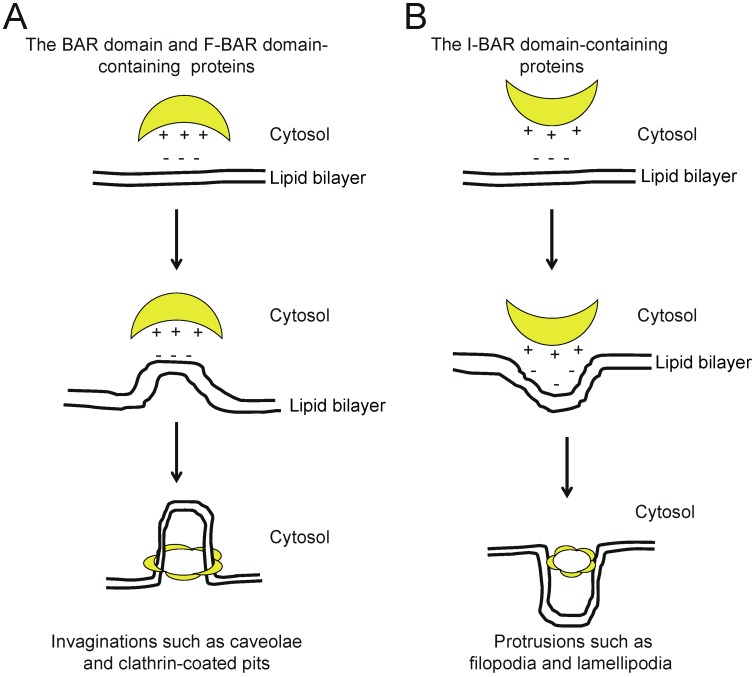
Schematic models for membrane deformation, based on the geometries of basic-charged amino acid residues that correspond to the structures of the membrane-binding surface of the F-BAR domain-containing proteins. (**A**) BAR or F-BAR proteins bind to the membrane to generate invaginations, such as caveolae and clathrin-coated pits; (**B**) I-BAR proteins deform the membrane to generate protrusions, such as filopodia and lamellipodia.

The BAR proteins are evolutionarily conserved, from yeast to human [1,2,3,4]. The BAR domains typically form a dimer, which is the unit for membrane binding. The structures of the BAR domain dimers resemble a banana. Each dimer possesses a distinct curved surface, from almost flat like Pinkbar to those of various steepness, where the basic-charged amino acid residues form a cluster, and deforms the membrane through the binding of the curved surface to the negatively-charged phospholipids in the plasma membrane (Figure 2). 

BAR proteins include subfamilies defined by their BAR, N-BAR/BAR (the Bin-Amphiphysin-Rvs167), F-BAR (extended Fes-CIP4 homology (EFC)/FCH-BAR), or I-BAR (IRSp53-MIM homology domain I-BAR/inverse-BAR) domains [1,2,3,4]. SH3 domains are commonly observed among the BAR domain superfamily proteins. Most of the SH3 domains bind to WASP family proteins and dynamin. The WASP family proteins include N-WASP and WAVE, which are activators of the Arp2/3 complex. The Arp2/3 complex mediates actin polymerization, which is involved in lamellipodia formation, podosome formation, clathrin-mediated endocytosis, pathogen infection and neurite extension downstream of N-WASP and WAVE [5]. Dynamin catalyzes membrane scission [6,7]. The SH3 domain also binds to several other molecules, such as the lipid phosphatase synaptojanin [8]. Synaptojanin has been linked to uncoating of clathrin-coated vesicles (CCVs) [8]. Therefore, the BAR domain superfamily proteins that are coupled to dynamin, WASP/WAVE family proteins, and other proteins are involved in several biological functions, including regulation of both the cytoskeleton and membrane shape. In this review, we describe the current information about each BAR protein with an SH3 domain(s). We also describe some BAR proteins that lack the SH3 domain, which regulate the actin cytoskeleton.

## 2. The BAR and N-BAR Domain Subfamily

**Figure 3:**
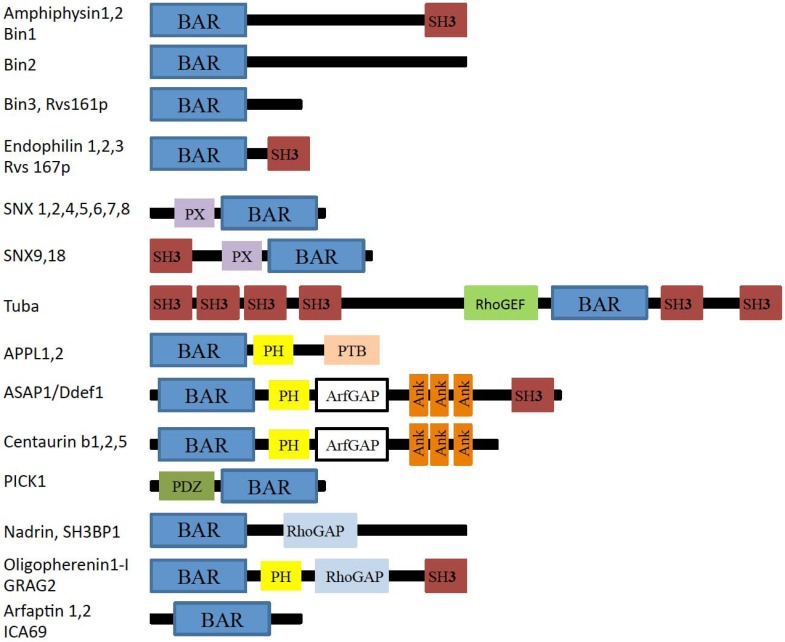
Domain organization of BAR subfamily proteins. BAR: Bin-Amphiphysin-Rvs domain; SH3: Src homology 3 domain; RhoGAP: Rho GTPase activating protein domain; PX: Phox homology domain; PH: Pleckstrin Homology domain; ArfGAP: Arf Rho GTPase activating protein domain; PTB: Phospho-tyrosine binding domain; PDZ: Psd-95, Dlg and ZO1 domain; RhoGEF: Rho guanine-nucleotide exchange factors domain; Ank: Ankyrin.

The BAR subfamily contains the members that do not belong to the F-BAR and I-BAR subfamilies. The N-BAR domain is included in the BAR subfamily that contains amino acids that form an amphipathic helix upon membrane binding in front of the BAR domain fold. This helix is thought to enhance membrane-binding ability by its insertion into the membrane [9,10]. The domain organization of the BAR protein subfamily is shown in Figure 3.

### 2.1. Amphiphysin

The structure of the BAR domain was first solved for Arfaptin, but was first characterized as a membrane-binding domain for amphiphysin [9,11]. The positively-charged surface of the amphiphysin BAR domain binds to the negatively-charged plasma membrane, mostly through phosphatidylserine and phosphatidylinositol (4,5)-bisphosphate [PI(4,5)P_2_] [9]. Furthermore, the BAR domains from amphiphysin have membrane-induced amphipathic helix on the dimer ends, and thus it is classified as an N-BAR domain. The hydrophobic amino acids are inserted into the membrane, like a wedge, thereby strengthening the interaction between the membrane and the BAR domain [9]. The SH3 domain of amphiphysin binds both dynamin and N-WASP, and these interactions are thought to be important for its function [12,13]. Amphiphysin has a binding site for clathrin between the BAR domain and the SH3 domain. Dynamin binding to the SH3 domain of amphiphysin was shown to disrupt clathrin binding to the N-terminal region [14]. Recruitment kinetics of amphiphysin and other BAR proteins to CCPs was recently shown by Taylor *et al*. [15]. The narrow tubular invaginations in the membrane, generated by the BAR domain of amphiphysin, suggested that amphiphysin acts at a late stage of fission for clathrin-mediated endocytosis. 

In addition to endocytosis, the N-BAR domain of amphiphysin is required for ruffle formation and phagocytosis, although its correspondence to the membrane curvature is unclear [16]. Phagocytosis is dependent on the activation of the Rho GTPase family proteins, such as Cdc42 and Rac, which regulate actin polymerization by the Arp2/3 complex and the WASP family [17,18]. N-WASP dimerization improves Arp2/3 binding to VCA domain, this can occur efficiently due to the dimeric/oligomeric nature of BAR proteins [19,20]. Therefore, amphiphysin might provide an additional layer of WASP regulation through its SH3-mediated binding. 

The membrane tubulation activity of amphiphysin *in vitro* was originally reported by Takei *et al*. [13]. Moreover, amphiphysin tubulation activity was also found in T-tubules [21]. T-tubules are invaginations of the external membranes of skeletal and cardiac muscle cells, which are rich in ion channels required for excitation-contraction coupling. Amphiphysin-2, a variant of the BAR protein, is highly expressed in skeletal muscle, and is localized on T-tubules [22]. Interestingly, this variant lacks the binding sites for clathrin and the clathrin adaptor AP-2, and instead comprises a polybasic sequence (encoded by exon 10) that enhances its affinity for the plasma membrane by electrostatic interactions [22]. Amphiphysin-2, in cooperation with other proteins, plays a critical role in the induction and stabilization of this unique organelle. Genetic disruption of the only *amphiphysin* gene in *Drosophila* disrupts the T-tubule network [21], and missense mutations in the human gene encoding amphiphysin-2 cause myopathies [23]. In addition, amphiphysin-2 (BIN1) tubulates membranes, either by itself or cooperatively with dynamin 2 (DNM2) [13,24]. The cooperation between BIN1 and DNM2 is mediated by the interaction of the BIN1 SH3 domain with the proline-rich domain of DNM2. However, this interaction may not occur prior to the association of BIN1 with membranes, since the polybasic sequence binds to the SH3 domain when it is not membrane-bound [25]. Indeed, PI(4,5)P_2_ binding is necessary to release the SH3 domain from the poly-basic region, enabling the interaction between the SH3 domain and DNM2. The existence of this intermolecular regulation was elucidated in cultured cells [25].

### 2.2. Endophilin

Endophilins are composed of an N-terminal BAR domain and a C-terminal SH3 domain. The BAR domain of endophilin A1 forms a wedge at the center of the BAR domain dimer, which is thought to be inserted into the membrane [10,26,27]. Endophilin participates in clathrin-dependent endocytosis via the BAR and SH3 domains as well as the uncharacterized “fast mode” of endocytosis [28]. The endophilin SH3 domain interacts with dynamin, N-WASP, and the phosphoinositide phosphatase synaptojanin [8,29]. A recent study suggests that Endophilin BAR domain dimerization on membranes triggers the access of ligands to the SH3 domain [30]. Recently, by using knock-out mice of all three endophilin A isoforms, it was demonstrated that all three endophilins, A1, A2, and A3, are involved in the recycling of synaptic vesicles at the uncoating stage of CCVs (clathrin-coated vesicles), rather than the scission of CCPs (clathrin-coated pits). This result is consistent with a role for Endophilin in Synaptojanin recruitment, not for dynamin recruitment or vesicle fission [8].

There are several possible links between diseases and endophilin A. Interestingly, it was recently reported that the SH3 domain of endophilin A binds with high affinity to Parkin, a protein linked to Parkinson’s disease [31], and also to huntingtin and ataxin-2, two additional proteins implicated in neurodegenerative diseases [32]. Endophilin 1,2 double knock-out mice develop neurodegerative disease leading to epileptic seizures [8].

In contrast to endophilin A, endophilin B1, also known as Bif1 or SH3GLB1, interacts via its N-BAR domain with Beclin 1, the mammalian homologue of yeast Atg6 (autophagy-related gene 6), through the UVRAG protein (UV irradiation resistance-associated gene). This interaction regulates the formation of autophagosomes, by promoting the activation of phosphatidylinositol (3) kinase C3 (PI3 kinase C3) [33]. Endophilin-B1 participates in the maintenance of mitochondrial morphology. The depletion of endophilin-B1, by using short-hairpin RNAs, leads to the dissociation of the outer mitochondrial membrane and the formation of vesicular and tubular structures from the remnants of this membrane [34]. These results were phenocopied by the knockdown of the dynamin homolog Drp1 (dynamin-related protein 1), a protein implicated in mitochondrial fission. Thus, Drp1 and endophilin-B1 may function in concert, and perhaps interact directly, in the maintenance of mitochondrial morphology [34]. Endophilin-B1 may also interact with Bax, Bcl2-associated X protein, to promote apoptosis following cytokine withdrawal [35]. However, in contrast to endophilin A1, there have been no reports of interaction of endophilin-B1 with N-WASP.

### 2.3. Sorting Nexins

Sorting nexins contain the BAR domain and the PX domain, but only SNX9 and 18 contain the SH3 domain. The BAR domain of the sorting nexin, SNX9, is in the proximity of the PX domain, which specifically recognizes phosphoinositides, such as PI(4,5)P_2_. The BAR and PX domains function as one unit with broad phosphoinositide specificity [36,37]. The roles of the BAR domains in the other SNX proteins have not been well studied. The PX domain shows affinity for various phosphoinositides, but it may also be involved in protein–protein interactions [38]. The BAR-PX unit of SNX9 also deforms membranes into tubules, and SNX9 is involved in clathrin-mediated endocytosis and endosomal trafficking [36,37,39]. SNX9 and the closely related SNX18 are accessory proteins required for the fission of clathrin-coated endocytic vesicles [40]. The SH3 domain of SNX9 binds to N-WASP and dynamin [39]. The SNX9 protein participates in the Arp2/3 complex activation by N-WASP, in the presence of liposomes [41].

SNX1 and SNX2 function in endosome trafficking. Although SNX1 and SNX2 lack the SH3 domain for N-WASP binding, another Arp2/3 complex activating protein, WASH, reportedly associates with SNX1 and SNX2 [35,36]. 

### 2.4. Tuba

Tuba contains the BAR domain, four N-terminal SH3 domains, a DH domain and two C-terminal SH3 domains. The four N-terminal SH3 domains exhibit strong affinity for dynamin, the DH domain is a Cdc42 GEF, and the C-terminal SH3 domains bind directly to the N-WASP and Ena/VASP proteins. Tuba is localized at synapses and dorsal ruffles. The membrane deformation induced by the BAR domain of Tuba has not been elucidated yet [42,43]. The role of Tuba in apical junction formation in epithelial cells dependent on Cdc42 and N-WASP is demonstrated [44]. Tuba is also shown to be required for epithelial cyst morphogenesis [45].

### 2.5. APPL1

APPL1, Centaurin, and ASAP1 are composed of the BAR and PH domains. Among the three proteins, ASAP1 also contains an SH3 domain (Figure 3). The PH domain is a module for phosphoinositide recognition. APPL1 is present on endosomal vesicles derived from clathrin-mediated endocytosis and on macropinosomes [46]. The dissociation of APPL1 from endosomes is reportedly correlated with the recruitment of PI(3)P binding proteins, such as WDFY2 and EEA1, to endosomes [47]. 

The BAR domain of APPL1 binds not only to lipids but also to small GTPases, using different surfaces. BAR domains of APPL1/2 interact with Rab5, which regulates endosomes maturation and fusion [48]. In addition, in response to extracellular stimuli, such as epidermal growth factor (EGF), APPL1 and 2 translocate from the membrane to the nucleus, where they interact with the nucleosome remodeling and histone deacetylase complex (NuRD/MeCP1) and thus regulate cell proliferation [49]. The release of the APPL proteins from endosomes and their subsequent translocation to the nucleus occur in a Rab5-dependent fashion, and GTP hydrolysis by Rab5 is required to release APPL [49]. 

### 2.6. ASAP1

ASAP1 is an ArfGTPase activating protein (GAP) containing a BAR domain. The PH domain of ASAP1 binds to PI(4,5)P_2_, which stimulates the GAP activity [46,50,51]. The proline-rich domain of ASAP1 binds to Src [52] and CrkL [53]. These Src and CrkL proteins also participate in the formation of the podosome. Podosomes facilitate cell migration and tissue invasion by immune cells. Invadopodia are related structures in invasive cancer cells. And ASAP1 is required for podosome formation in NIH3T3 cells. Furthermore, the Src-dependent phosphorylation of ASAP1 on Tyr-782 is necessary for podosome formation [51]. The SH3 domain of ASAP1 can also bind to cortactin and FAK [51], although interactions with N-WASP and dynamin were not reported. However, another study showed that ASAP1 interacts through its BAR domain with the C-terminal region of GEFH1, a guanine nucleotide exchange factor for RhoA, and thus inhibits podosome formation [54]. Therefore, further studies are required to clarify the role of ASAP1 in podosome formation.

ASAP3, which is closely related to ASAP1, is associated with focal adhesions and circular dorsal ruffles, but is not localized to podosomes [55]. The reduction of ASAP3 expression results in fewer actin stress fibers, reduced levels of phosphorylated myosin, and slower cell migration and invasion. Conversely, the down-regulation of ASAP1 has no effect on either migration or invasion [56,57].

### 2.7. PICK1

PICK1 contains a PDZ domain, but lacks an SH3 domain. The BAR domain of PICK1 reportedly interacts with the Arp2/3 complex to suppress the nucleation of actin filaments, thereby inhibiting the endocytosis of neurotransmitter receptors. The PDZ domain of PICK1 interacts directly with lipid membranes containing phosphoinositides, and the PDZ–lipid interaction is necessary for synaptic transmission [58,59,60]. The interplay between the BAR and PDZ domains of PICK1 has been reported [61]. Experimental observations suggested that the PICK1-PDZ domain inhibits the activity of the PICK1-BAR domain, and this auto-inhibition can be released by PICK1-PDZ ligand binding [62,63].

## 3. F-BAR Domain Subfamily

**Figure 4:**
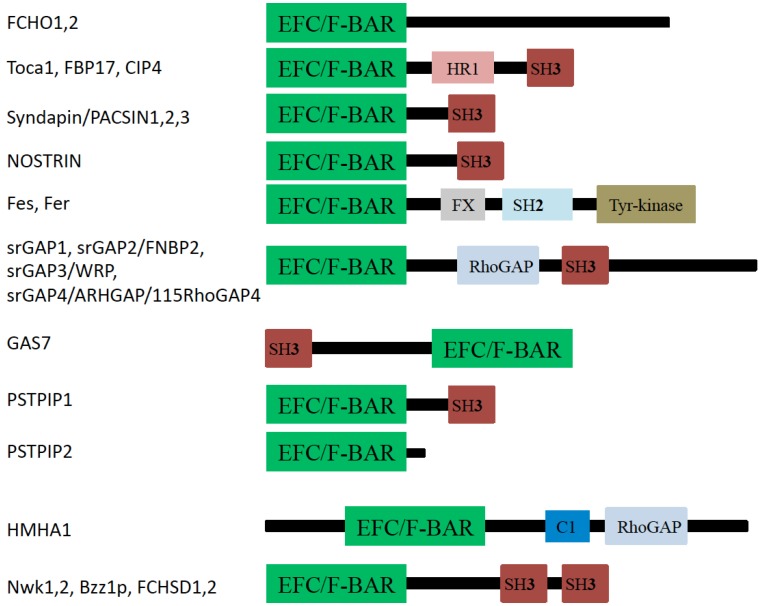
Domain organization of F-BAR proteins. EFC: extended FER-CIP4 homology (EFC) or FCH and BAR (F-BAR) domain; HR1: Protein kinase C-related kinase homology region 1 domain; SH2: Src homology 2 domain; SH3: Src homology 3 domain; FX: F-BAR extension domain; Tyr-kinase: Tyrosine kinase domain; RhoGAP: Rho GTPase activating protein domain; C1: Protein kinase C conserved region 1.

The F-BAR-domain-containing proteins exist in all eukaryotes, except plants. These proteins are also known as Pombe/Cdc15 homology (PCH)-family proteins, from the founding member of this family (Figure 4).

### 3.1. FCHo1 and FCHo2

The F-BAR domain of FCHo2 also forms a crescent-shaped dimer, but the curvature of its membrane-binding, concave surface is smaller than those of the F-BAR domains of CIP4 and FBP17 [64]. Correspondingly, the FCHo1/2 proteins were required for the initiation of clathrin assembly at the plasma membrane through binding to the scaffold proteins for clathrin-coated pits, such as eps15 and intersectin, which in turn recruit the adaptor complex AP2 for clathrin assembly [65]. 

### 3.2. FBP17, CIP4, and Toca-1

The structures of the F-BAR domain from CIP4 and FBP17 were the first to be solved, among the F-BAR domains [66]. The amino acid sequence of the F-BAR domain of Toca-1/formin binding protein 1-like (FNBPIL) is almost identical to those of CIP4 and FBP17 [66,67,68]. Toca-1 was identified biochemically as an essential component of Cdc42-mediated actin polymerization [69]. The diameter of tubules generated by the F-BAR domains of CIP4 and FBP17 is much larger than that induced by the BAR domains of amphiphysin and endophilin, and appears to correspond to the curvature of the initial stages of clathrin-coated pits [66,67,68]. An isomeric variant of CIP4, CIP4h (also known as CIP4/2) was identified, and both CIP4h and CIP4 reportedly function directly in the translocation of GLUT4 during endocytosis [70,71]. CIP4 regulates insulin signaling downstream of TC10 GTPase, via recruitment of GAPex-5 to inactivate Rab31 leading to increased GLUT4 vesicle trafficking to the cell surface [72]. However, CIP4 was shown to inhibit GLUT4 trafficking to the cell surface using both knock-down and knock-out approaches [71,73]. Resolving these conflicting findings may require resolving CIP4’s role in endocytosis and exocytosis of GLUT4.

The SH3 domains of FBP17 and CIP4 bind to dynamin, which antagonizes the tubulation activities of FBP17 and CIP4 [67,68]. The SH3 domain also binds to N-WASP, which functions downstream of Cdc42. In addition to the F-BAR domain, FBP17, Toca-1 and CIP4 possess Cdc42, Rnd2 and/or TC10 binding sites (HR1 domain: homology region 1) [69,70,74,75]. Toca-1, FBP17, CIP4, and Cdc42 are involved in regulating the activation of N-WASP [67,68,69,76]. CIP4 reportedly associates with the proline-rich domain of Wiskott-Aldrich syndrome protein (WASP) in macrophages and CIP4 can regulate localization of WASP in macrophages [77,78].

FBP17, CIP4, and Toca-1 are involved in a variety of structures, in addition to clathrin-coated pits. The F-BAR and SH3 domains of FBP17 are essential for the formation of podosomes and phagocytic cups in macrophages [79]. The microinjection of a CIP4 mutant lacking its SH3 domain resulted in the inactivation of podosome formation in macrophages, suggesting a critical role for the CIP4/WASP interaction [80]. Moreover, the F-BAR and SH3 domains of FBP17 are necessary for recruiting the WASP-WIP complex and dynamin-2 to the plasma membrane [67,79,81,82]. In macrophages, FBP17 interacts with the WASP-WIP complex and dynamin-2 to form podosomes, through its F-BAR and SH3 domains. Therefore, complex formation between the F-BAR domain of FBP17 and PI(4,5)P_2_ may be required for the membrane localization of FBP17 as well as the WASP-WIP complex and dynamin-2, to determine where podosomes will form [79]. 

CIP4 localization to invadopodia was reported in breast cancer cells [83]. Notably, CIP4 is involved in the surface expression of transmembrane type 1 matrix metalloprotease (MT1-MMP), an important metalloprotease for matrix degradation at the podosome. CIP4 is reportedly phosphorylated by Src at the linker region between the HR1 domain and the SH3 domain [84]. Tyrosine phosphorylation at Y471 by Src is important for matrix degradation as well as invasion in breast tumor cells [84]. Src kinase inhibits endocytosis of MT1-MMP to promote cell invasion, which is partly explained by phosphorylation of BAR proteins [85], first shown by Wu *et al.* (2005) to involve disruption of Dynamin binding to Endophilin following SH3 domain phosphorylation by Src. This observation is consistent with CIP4 phosphorylation by Src to reduce MT1-MMP internalization [84,85].

Although Toca-1 is thought to have a concave membrane-binding surface, the role of F-BAR domain-containing proteins, such as Toca-1, in filopodia formation has been suggested by reconstitution assay [86]. This might occur through membrane binding to the neck region of filopodia, in a similar manner to PACSIN, as described in the next section [86,87]. CIP4 also reportedly functions in lamellipodia, although the mechanism is unclear [88]. Knock-out mice experiments revealed that CIP4 is essential for optimal GC (germinal center) formation, skin inflammation, and integrin-dependent T-cell migration [89].

Toca-1 promotes vesicle motility, filopodia and lamellipodia formation by recruiting N-WASP or Abi1, respectively [90,91,92]. Toca-1/FNBPIL is essential for autophagy of the intracellular pathogen *Salmonella enterica* serovar Typhimurium [93]. The interaction between Toca-1 and ATG3 (autophagy protein) occurs through the Toca-1 HR1 domain [93]. Moreover, CIP4 was proposed to be involved in endosomal trafficking [94]. 

In terms of their relationships to diseases, the SH3 domains of CIP4 bind to the huntingtin protein, which is mutated in patients with Huntington disease [95]. CIP4 has been implicated in renal cancer, where a mutation causes the expression of a truncated CIP4 fragment including the F-BAR domain and lacking the SH3 and HR1 regions [96]. Recent studies implicate CIP4 family proteins in cancer cell invasion [79,83,84,92], and will require extension to tumor metastasis studies in animal models.

### 3.3. PACSIN (Syndapin)

The syndapins (synaptic dynamin-associated proteins), also known as PACSINs (PKC and casein kinase substrate in neurons), have three isoforms. Whereas PACSIN1 is restricted to neurons, PACSIN2 is ubiquitously expressed and PACSIN3 is present in lung and muscle tissues [97]. PACSINs have been implicated in the regulation of both clathrin-mediated endocytosis [98,99] and caveolae endocytosis [100,101]. PACSINs contain an N-terminal F-BAR domain and a C-terminal SH3 domain. The F-BAR domains of PACSINs have a hydrophobic wedge [102,103]. Overexpression of the SH3 domain of syndapins inhibits receptor-mediated endocytosis [104].

Structurally, PACSINs have a concave membrane-binding surface, as in the other F-BAR domains, such as FBP17, CIP4, and FCHo2 [102,103]. Therefore, the inward membrane tubulation is a natural outcome of its membrane binding. The membrane tubules induced by the PACSIN F-BAR are narrower than those induced by the F-BAR domains of FBP17 and CIP4, which correlates with the structural differences between these proteins [100,103]. The narrower diameter of the tubules suggests that PACSIN2 is involved with the clathrin-coated pit at a transient, late stage of clathrin-coated vesicle fission. PACSIN2 is also localized at caveolae, which typically have a narrower neck diameter than clathrin-coated pits [100,101]. The direct binding of the PACSIN2 F-BAR domain with caveolin-1 supports the role of PACSIN2 in caveolae. 

However, overexpression of the full-length protein generates microspikes and lamellipodia-like structures, as well as cellular invaginations [102,105]. For protrusions such as microspikes, the concave membrane-binding surface might fit to the neck of protrusions, where the same positive curvatures were found. The PACSIN2 F-BAR domain alone appears to be localized at these necks [102].

The SH3 domain of PACSIN2/syndapin II also binds to N-WASP and dynamin [98]. The SH3 domain seems to contribute to the auto-inhibition of PACSIN1’s membrane tubulation ability. The basic patch on the F-BAR domain interacts with a corresponding acidic surface on the PRD-binding RT loop of the SH3 domain [106]. Interestingly, such charge complementarity is also used by the PxxP motifs within SH3 domain ligands, such as dynamin1 [107]. On the other hand, the intermolecular interaction between F-BAR domains can compete with the intramolecular SH3/F-BAR interaction [106]. These intramolecular interactions have recently been observed with BAR domain-containing proteins Endophilin, where Endophilin BAR domain dimerization on membranes is suggested to trigger the access of ligands to the SH3 domain [30]. Thus, perhaps the SH3 domain does not inhibit the BAR domain, but this curvature sensing domain triggers the appropriate subcellular space to engage with its ligand. In addition, the SH3 domains of PACSIN1 bind to huntingtin protein, which is mutated in patients with Huntington disease [108].

### 3.4. NOSTRIN

NOSTRIN contains an F-BAR domain and an SH3 domain, and is reportedly localized at caveolae. The SH3 domain of NOSTRIN binds to N-WASP and dynamin [109,110]. However, the structure of the NOSTRIN F-BAR domain has not been reported. Patients with alcoholic hepatitis had significantly high hepatic levels of NOSTRIN. NOSTRIN induces the intracellular translocation of endothelial NO synthase (eNOS) and reduces NO generation, indicating that NOSTRIN expression is regulated under pathologic conditions [111].

### 3.5. Fes and Fer

The Fes/Fps and Fer proteins are a distinct family of non-receptor tyrosine kinases, with prominent roles in inflammation and immunity [112,113,114]. Fes/Fps and Fer have a kinase region, an SH2 domain, an F-BAR extension (FX) domain and an F-BAR domain. The kinase region resembles those of the Src family tyrosine kinases. The region adjacent to the Fes or Fer F-BAR domain binds to phosphatidic acid (PA), and was named the FX domain. The F-BAR and FX units act as a membrane binding module with a preference for PA, and the F-BAR and FX units are essential for the membrane-dependent activation of the Fer kinase activity, which is involved in lamellipodia formation and cell migration [115]. The structure of the F-BAR-FX unit has not been determined. Recent study shows that Fes not only binds PA, but to phospholipase D2 (PLD2), leading to increased differentiation of myeloid leukemia cells [116] . Fer was localized to microtubule ends, and it can phosphorylate the adhesion molecule platelet/endothelial cell adhesion molecule 1 (PECAM-1) [117]. Fes signaling in stromal cells promotes breast tumor growth and metastasis [118]. Fes expression was also recently shown to have prognostic value for recurrence of prostate cancer [119]. Fer was recently linked to resistance of lung adenocarcinoma cells to quinicrine, an anti-malarial drug with anti-cancer properties [120]. Fer and a truncated Fer isoform (FerT) have recently been implicated in promoting growth and survival in colon cancer cell lines [121].

### 3.6. srGAPs

The slit-robo GAP proteins, (srGAP)1–4, contain an F-BAR domain at the N-terminus, a RhoGAP domain in the middle and an SH3 domain at the C-terminus [122,123]. The specificity of the GAP activity on small GTPases differs among the srGAP1–4 proteins. The SH3 domain often binds to WASP/WAVE proteins. srGAP1 binds to WASP, and inactivates Cdc42. In this respect, *srgp-1* (nematode ortholog of mammalian srGAP) senses membrane invaginations through its BAR domain, and is involved in cell corpse clearance and sick-cell killing in *C. elegans* [124]. srGAP2 binds to N-WASP and inactivates Rac [123,125]. srGAP3/WRP binds to WAVE1, and inactivates Rac [122]. The overexpression of the F-BAR domain-containing fragment of srGAP2 induced filopodia-like protrusions without actin filament localization, in a similar manner to the overexpression of the I-BAR domain of IRSp53. However, its membrane-binding mechanism is still unclear [123]. A recent mutational analysis revealed that the predicted concave surface does not bind membranes [126]. The srGAP3 gene is deleted in a severe type of mental retardation [127].

### 3.7. GAS7

GAS7 contains the SH3 domain and the F-BAR domain. The SH3 domain is located at the N-terminus of the F-BAR domain, and this is the unique characteristic of GAS7. The structure of the F-BAR domain is unknown. The SH3 domain binds to N-WASP [128]. GAS7 plays a role in neuronal development [128], and its ability to cross-link actin and modulate actin dynamics can induce cell protrusions [129]. 

### 3.8. PSTPIP1/2 and Cdc15

PSTPIP1 was first identified as a tyrosine phosphorylated protein associated with F-actin [130]. PSTPIP1 contains an F-BAR domain and an SH3 domain, and the latter domain interacts with WASP and a tyrosine phosphatase [131]. The structure of its F-BAR domain is unknown. Mutations in the PSTPIP1 gene cause a rare autoinflammatory disease (PAPA syndrome). Two mutations (E250Q and A230T) were found in the BAR domain of patients with PAPA syndrome, although the effects of these mutations on PSTPIP1 function have not been clarified [132]. 

PSTPIP2 shares about 41% sequence homology with its counterpart, PSTPIP [131]. PSTPIP2 has only the F-BAR domain, and no other domain was identified. Two sites of tyrosine phosphorylation and a binding site for PTP-PEST are found in both PSTPIP1 and PSTPIP2 [131]. Recently, PSTPIP2 has been linked to filopodium formation, through its putative F-actin bundling activity [133]. However, the role of the membrane-binding F-BAR domain in filopodium formation has yet to be investigated. It was shown that PSTPIP2 regulates the organization of the actin cytoskeleton, as well as macrophage morphology and motility, in response to Colony Stimulating Factor-1 (CSF-1) [133]. PSTPIP2 interacts with F-actin and increases cortical actin bundling. *In vitro*, PSTPIP2 induces F-actin bundling, decreases the actin polymerization rate, and increases F-actin stability [133]. Moreover, PSTPIP2 reportedly binds phospholipids and deforms the plasma membrane into narrow tubes in COS-7 cells [67]. In whole animals, the anti-inflammatory role of PSTPIP2 was shown in mouse model studies [134,135]. Mice with PSTPIP2 mutations that cause reduced expression levels (cmo and lupo) leads to fatal autoimmune disease due in part to hyperactivation of macrophages [136].

PSTPIP1 is localized to the cleavage furrow of cultured human cells [137]. PSTPIP1 is highly homologous to *Saccharomyces cerevisiae* Cdc15, but lacks the long linker region between the F-BAR domain and the SH3 domain present in Cdc15. Cdc15 is localized at the contractile ring and is essential for cytokinesis [138,139,140]. The phosphorylation of Cdc15 controls its function in cytokinesis. On the other hand, phosphorylation at many sites within Cdc15 generates a closed conformation, which inhibits Cdc15 assembly at the division site in interphase. Conversely, Cdc15 dephosphorylation induces an open conformation, oligomerization, and scaffolding activity during mitosis [141]. Cdc15 was described originally as an SH3 domain-containing protein that regulates actin nucleation, through the recruitment of formin Cdc12 and type I myosin Myo1 to the contractile ring by its F-BAR domain [142]. Cdc15 also stabilizes the contractile ring through its SH3 domain [143]. 

F-BAR domains are also found in various other *Saccharomyces cerevisiae* proteins such as Cyk2/Hof1, Bzz1 and Rgd1–2 and *Schizosaccharomyces pombe* proteins such as Imp2, YB65 (pombe Bzz1p) and Rga7–9 [144,145]. Hof1 is localized at the site of cell division where septin is present. In addition, the deletion of the F-BAR domain of Hof1 reportedly caused defective actomyosin ring contraction [146].

## 4. I-BAR Subfamily

**Figure 5:**
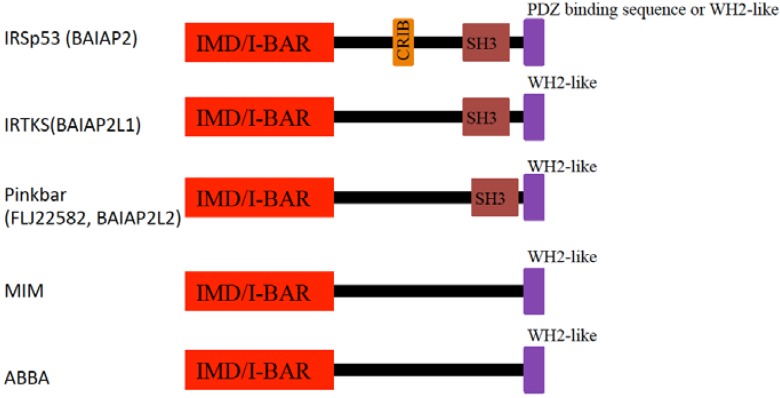
Schematic of domain organization of I-BAR proteins. IRSp53-MIM homology domain (IMD)/inverse-BAR (I-BAR); CRIB: Cdc42-Rac interactive binding region; SH3: Src homology 3 domain; WH2: Wasp homology 2 (verprolin homology) domain.

The I-BAR domain binds to the membrane through its convex surface. The I-BAR domain, with the inverted geometry of the membrane-binding surface, as compared to most BAR and F-BAR domains, is involved in the plasma membrane protrusions of filopodia and lamellipodia. The structures of the I-BAR domains from IRSp53, MIM, and Pinkbar have been determined [147,148,149,150] (Figure 5).

### 4.1. IRSp53

*In vitro*, the incubation of the I-BAR domain with liposomes induced membrane invaginations, which corresponded geometrically to cellular protrusions [148]. The binding of the I-BAR domain to these membrane structures was confirmed by cryo-electron microscopy [151]. The I-BAR interaction with the membrane occurs through phosphatidylserine, but a preference for PI(4,5)P_2_ and PI(3,4,5)P_3_ was observed for the I-BAR of IRSp53 [148,149,151]. The overexpression of the I-BAR fragment alone induced highly dynamic membrane protrusions that even persisted in the presence of an actin polymerization inhibitor [148,149,152,153]. Furthermore, several regions without actin filaments were also observed in these protrusions [148,154]. When the full-length proteins were overexpressed, the induced protrusions contained actin filaments, presumably because the SH3 domain recruits proteins that bundle and/or induce the formation of actin filaments. Interestingly, the SH3 domain of IRSp53 binds to the Arp2/3 activator, WAVE2, which plays essential roles in lamellipodium formation, and also to N-WASP, which is considered to function in filopodium formation and endocytosis [155,156,157]. The SH3 domain also binds to MENA and VASP, which promote actin polymerization without branching [157,158,159]. The IRSp53 SH3 domain reportedly binds to dynamin [157], but the significance of dynamin in membrane protrusions is still unclear. Several laboratories have confirmed I-BAR binding to the actin filament, though its significance in relation to membrane deformation is unclear [147,148,149]. IRSp53 also has a PDZ binding motif, and its interactions with several PDZ domain-containing proteins may be important for the assembly of some cellular structures [160,161,162,163].

In cells, IRSp53 is involved in both filopodium and lamellipodium formation, as suggested from the localization and the binding of WAVE2, N-WASP, VASP, and Mena [155,156,158,159]. Filopodia are regulated by the small GTPase, Cdc42. IRSp53 contains a Cdc42-binding motif and seems to be required for the Cdc42-induced formation of filopodia [157]. This latter activity might also depend on its association with another cytoskeletal modulator, epidermal growth factor receptor kinase substrate 8 (Eps8), which is known to function in actin-capping and -bundling [164]. In addition, an analysis with N-WASP knockout cells indicated that the IRSp53-mediated formation of filopodium-like protrusions requires N-WASP, but its Arp2/3 complex activating ability was not involved in the protrusion formation, and thus IRSp53 might be sufficient for unbranched actin filament formation in filopodia [157]. 

The siRNA-mediated knockdown of IRSp53 also revealed its role in lamellipodia formation [156]. Moreover, IRSp53 is required, in association with the WAVE2-Abi1 complex, for various actin-mediated processes such as lamellipodium formation, but not for the formation of filopodia [165]. The lamellipodia-like structures induced by WAVE2 and IRSp53 are involved in phagocytosis [165,166]. In *Dictyostelium discoideum*, the IBARa protein (which contains an I-BAR domain) is involved in curvature sensing, and its SH3 domain recruits regulators of actin polymerization, including the Arp2/3 complex, at the neck of a particle during phagocytosis [167]. 

### 4.2. MIM

MIM and ABBA lack an SH3 domain. Both MIM and ABBA are composed of a C-terminal actin-monomer binding WH2 (WASP homology 2) domain and an N-terminal I-BAR domain. The WH2 domain of MIM directly binds to actin [168,169]. In addition to its induction of a negatively curved membrane, the I-BAR from MIM also has a wedge for insertion into a membrane enriched in PI(4,5)P_2_, which it then deforms into tubular structures *in vitro* [148,149,151]. MIM enhances Arp2/3-mediated actin polymerization through interactions with cortactin, but inhibits WASP-mediated actin polymerization [169]. 

MIM is strongly expressed during development in muscles and postmitotic neurons, and in adult mice in the kidneys, liver, and Purkinje cells of the cerebellum [168,170]. Mouse model studies revealed that MIM deficiency leads to a progressive kidney disease characterized by abnormal tubular morphology, severe urine concentration defects, renal electrolyte wasting and bone abnormalities [171]. Interestingly, MIM was recently also implicated in the Sonic hedgehog (Shh) signaling pathway. Shh is a potent morphogen that controls many developmental processes, including left–right asymmetry and organ patterning. Ectopic or dysfunctional Shh signaling has been linked to many cancers [172].

### 4.3. Pinkbar

Pinkbar (planar intestinal- and kidney-specific BAR domain protein) contains an I-BAR domain and an SH3 domain. The I-BAR domain of Pinkbar is noticeably shorter (164 Å) than those of IRSp53 (182 Å) [147] and MIM (185 Å) [173]. The I-BAR domain of Pinkbar interacts with PI(4,5)P_2_-rich vesicles through electrostatic interactions. Due to its flat structure, the BAR domain of Pinkbar does not induce membrane protrusions or invaginations, and instead deforms phosphoinositide-rich membranes into planar structures [150]. The binding partner for the SH3 domain has not been identified.

Pinkbar is expressed predominantly in intestinal and kidney epithelial cells, where it localizes to Rab13-positive vesicles and to the plasma membrane at the cell-cell junctions [150]. The small GTPase, Rab13, is highly expressed in the intestinal epithelial cells and promotes tight junction integrity [174,175]. Therefore, Pinkbar may be involved in the formation of specific membrane structures at the intercellular junctions of enterocytes that may regulate intestinal permeability or nutrient absorption. 

## 5. Conclusions

We have summarized the current information available for the well-studied BAR proteins. More than 50 different types of BAR proteins are present in humans, and therefore we still lack a complete understanding of the membrane curvature generation and sensing mediated by BAR proteins. Several BAR proteins are associated with multiple structures, and thus the BAR proteins may simply function for the generation and recognition of membrane curvature, and might not have one-to-one relationships to subcellular structures. The various combinations of BAR proteins for each subcellular structure may provide its characteristic membrane shape.
